# 

**Published:** 1843-01

**Authors:** 


### WESTERN JOURNAL

OF

### MEDICINE AND SURGERY.

### EDITORS.

---

DANIEL DRAKE, M. D.,

PROFESSOR OF PATHOLOGICAL ANATOMY AND CLINICAL MEDICINE IN THE MEDICAL INSTITUTE OF LOUISVILLE:

LUNSFORD P. YANDELL, M. D.,

PROFESSOR OF CHEMISTRY AND PHARMACY IN THE SAME:

THOMAS W. COLESCOTT, M. D.

### RECEIPTS OF THE MEDICAL JOURNAL.

### FOR THE MONTHS OF NOVEMBER AND DECEMBER.

| | |
|---|---|
| Dr. W. M. Mixon, Spring Hill, Miss., | $5 00 |
| H. Alexander, Palestine, Ill., | 5 00 |
| A. E. Geoghegan, Litchfield, Ky., | 5 00 |
| C. B. Walworth, Vernon, O. (discount $1,) | 5 00 |
| J. B. McGill, Ovid, O., | 5 00 |
| —— Christy, Farmington, Ill., | 5 00 |
| R. A. Armstead, Cadiz, Ky., | 5 00 |
| W. Girdner, Cedar Creek, Teen., | 10 00 |
| J. S. Athon, Charleston, Ia. | 5 00 |
| S. C. Bridgewatet, Clinton College, Tenn., | 5 00 |
| S. B. Robertson, Murfreesboro, Tenn., | 5 00 |
| —— Embree. Pine Bluff, Ark., | 5 00 |
| W. J. J. Morrow, Chattanooga, Tenn., | 10 00 |
| J. Fithean, Oxfore, O., | 5 00 |
| J. Penington, Cambridge, Ia., | 8 00 |
| J. Roberts, Montgomery, Ia., | 5 00 |
| S. T. Sharp, Cambridge, Ia., | 5 00 |
| C. H. Preston, Paris, O., | 3 00 |
| D. A. Kinchloe, Bellmont, Miss. | 5 00 |
| Drs. Barker and Packard, Keosonqua, Iowa., | 5 00 |
| Dr. P. Herrod, Pleasant Shade, Tenn., | 5 00 |
| J. C. Taylor, County, | 5 00 |
| J. N. Smith, Paris, Ky., | 5 00 |
| T. P. Massie, Auburn, Mo., | 5 00 |
| —— Crooks, Greenville, S. C. | 2 50 |

---

☞ C. W. James, of Cincinnati, Ohio, is authorised to collect the accounts of this Journal, in Ohio, Indiana, and Michigan. He will be assisted by M. Meeker, Jas. R. Smith, J. B. Humphrey, and J. T. Dent, all of whose receipts will be good.

PRENTICE & WEISSINGER.

---

The Western Journal of Medicine and Surgery is published monthly by the undersigned, at the corner of Main and Fifth streets, Louisville, at $5 per annum, payable in advance. Each number contains from 80 to 84 pages making two volumes in the year of about 500 pages.

Contributions to its pages by the Physicians of the Valley of the Mississippi, addressed to the Editors, are respectfully solicited.

Letters on business, to be addressed, postage paid, to the publishers.

☞Postmasters, by the regulations of the Postoffice Department, will frank letters containing subscription money, and all remittances so franked are at the risk of the publishers.

*August*, 1842. PRENTICE & WEISSINGER.

